# A46 BIOGEOGRAPHIC VARIATION AND FUNCTIONAL PATHWAYS OF THE GUT MICROBIOTA IN CELIAC DISEASE

**DOI:** 10.1093/jcag/gwab049.045

**Published:** 2022-02-21

**Authors:** J Libertucci, M Constante, H J Galipeau, J C Szamosi, G H Rueda, M I Pinto-Sanchez, C M Southward, L M Rossi, M E Fontes, F G Chirdo, M Surette, P Bercik, A CAMINERO FERNANDEZ, E Verdu

**Affiliations:** 1 Farncombe Family Digestive Health Research Institute, Hamilton, ON, Canada; 2 Universidad Nacional de la Plata, La Plata, Argentina

## Abstract

**Background:**

Genes and gluten are necessary, but insufficient to cause celiac disease (CeD), as risk alleles (DQ2 or DQ8) are prevalent in ~30–40% of the healthy population consuming gluten. Gut microbiota shifts and infections have been proposed as risk modulators. Biogeographic characterization of the microbiota in CeD patients and its functional significance are limited, particularly at the duodenum, the main site of inflammation.

**Aims:**

We studied microbiota composition and predicted function along the gastrointestinal tract and investigated the impact of host genetics and CeD activity.

**Methods:**

We used 16S rRNA gene sequencing (Illumina) and predicted gene function analysis (PICRUSt2), to study the microbiota in duodenal biopsies (D1, D2 and D3), duodenal aspirates, and fecal samples from patients with active CeD (n= 24) (biopsy and serology confirmed) and controls (non-celiac, n= 41). CeD alleles were determined in consented participants using DQ-CD typing. Small intestinal samples from controls (DQ2^-/-^ = 14; DQ2^+/-^ = 7) and CeD (DQ2^+/-^ = 12) were used for further analysis and to colonize C57BL/6 germ-free mice for gluten metabolism studies.

**Results:**

Microbiota community composition and predicted function was mainly determined by intestinal location (P= 0.001). Within the duodenum, but not in stool, CeD patients had increased abundance of opportunistic pathogens. *Escherichia coli* was increased in D1, *Streptococcus pneumoniae* in D2, and *Neisseria* in D3 versus controls. Predicted bacterial protease and peptidase genes were altered in CeD DQ2^+/-^ patients versus DQ2^-/-^ controls. In DQ2^+/-^ controls, fewer predicted bacterial genes were altered compared to CeD DQ2^+/-^ patients. Impaired capacity to metabolize gluten was confirmed in germ-free mice colonized with microbiota from CeD (DQ2^+/-^), but not DQ2^+/-^ or DQ2^-/-^ controls.

**Conclusions:**

In the duodenum, CeD is associated with increased opportunistic pathogens and altered bacterial proteolytic profile. These are not determined by genetic predisposition, as CeD and controls with similar genetic background differed in its predicted bacterial proteolytic function, which was confirmed in mice colonized with duodenal microbiota using these cohorts. Our study highlights the need for defining sampling location in studies investigating the role of microbiota in CeD.

**Funding Agencies:**

CAG, CCC, CIHR

